# Not Hungry, but Still Snacking: The Association Between Hedonic Hunger and Snacking Behaviour Among Young Adults in Vadodara, Gujarat

**DOI:** 10.7759/cureus.44814

**Published:** 2023-09-07

**Authors:** Margi Mankad, Devaki Gokhale

**Affiliations:** 1 Nutrition and Dietetics, Symbiosis Institute of Health Sciences, Symbiosis International (Deemed University), Pune, IND

**Keywords:** india, adolescents, eating habits, snacking behaviour, hedonic hunger

## Abstract

Background: Hedonic hunger refers to the recurring thoughts, desires, and feelings about food in the absence of energy deprivation. The influence of hedonic hunger on snacking behaviour remains unclear, particularly among young adults in India.

Aim: The present study aimed to understand the association between hedonic hunger and snacking behaviour among young adults aged 15-24 years in Vadodara, Gujarat.

Methods: The study utilized a cross-sectional design. A total of 150 young adults from Vadodara, Gujarat, were included in the study. Participants were administered a structured questionnaire. Hedonic hunger scores were measured using the Power of Food Scale assessment. Anthropometric assessment was carried out using validated instruments. The associations between hedonic hunger, snacking behaviour, emotional eating, willpower, exercise, weight, and Body Mass Index (BMI) were examined.

Results: Results showed that 51.3% of individuals had moderate levels of hedonic hunger, while 23.3% exhibited severe signs, indicating a strong inclination towards consuming appetizing foods rich in salt, sugar, and fat. The study found a significant association between hedonic hunger and consumption of certain snacking items. Furthermore, hedonic hunger scores were related to additional factors such as emotional eating, willpower, and exercise. Interestingly, a positive correlation was observed between hedonic hunger scores and participants' weight (r=0.170; P*=* 0.039), but no such correlation was found with BMI.

Conclusion: These findings shed light on the relationship between hedonic hunger, snacking behaviour, and related factors, contributing to our understanding of the complex interplay between food cravings and eating habits among adolescents.

## Introduction

The rising incidence of overweight/obesity in combination with undernutrition is a major challenge in low-income and middle-income countries. The prevalence of sedentary lifestyles, dietary changes, and urbanization have led to an increase in overweight and obese individuals. The presence of underweight along with overweight/obesity is defined as the double burden of malnutrition and is a major health challenge in developing countries like India [1]. Currently, India also marks an increasing adaptation towards a Western diet which mainly comprises a high intake of meat, high-fat dairy products, refined foods, and fast foods. India is in the middle of a nutrition transition; there is a shift in diet patterns from a diet high in cereals and fibre to a diet high in refined sugars, fats and animal food sources leading to an increase in obesity and chronic diseases [2]. An obesogenic environment is one that is not conducive to weight loss and promotes weight gain. Based on the following findings, there is a possibility that an obesogenic environment can stimulate hedonic hunger. 

Hedonic hunger can be characterized as an Individual’s obsession with food and the need to consume it for the purpose of enjoyment and in the absence of physiological hunger [3]. Since ancient times the primary motive behind eating was to survive by maintaining energy homeostasis levels and avoiding starvation. Owing to the transition of changing lifestyle, unhealthy dietary habits, and the presence of an obesogenic environment, the majority of the food consumption in today’s world occurs for reasons other than energy deprivation, giving rise to “non-homeostatic” eating or “hunger for pleasure” [4]. A food-abundant environment is characterized by a large number of low-cost, easily available, and palatable energy-dense foods that are always universal [5]. It is possible that hedonic hunger can affect eating patterns over homeostatic eating activity, which means eating more in response to hedonic aspects of food [6]. 

The psychobiological system consists of hedonic and homeostatic characteristics of hunger which work mutually and affect the overall food consumption. Neurobiological studies conducted in both animals and humans show that when a palatable food commonly high in sugar/salt/fat is consumed, it increases the activation in the reward-related areas of the brain which in turn causes the release of dopamine. The levels of dopamine secreted are related to the feeling of happiness that is obtained after consuming it. The oral, sensory, and gustatory properties of palatable food like smell and taste can also play a role in dopamine release [7].

Hedonic hunger can be assessed using the power of food scale (PFS) which estimates appetite and not the palatable food consumption. It consists of three domains which include the availability of food, the presence of food, and how the food tastes, and it is now considered a new measure of appetite [4]. With constant access to palatable foods all around, multiple eating habits and patterns occur throughout the day [8]. Three major meals (breakfast, lunch, and dinner) are consumed throughout the day, and the type of food that is consumed between any meals is referred to as “Snack” [9]. Snacking may contribute to an increase in energy intake and weight gain in various ways. Snacking contributes almost 30% of total energy intake. Studies conducted in the past have shown that snacking or consuming foods in between meals can be more affected by food-related signals. According to Hagmann, 2019, individuals with high levels of hedonic hunger have more processing in the visual regions of the brain in reaction to words and images that depict highly palatable foods and are more likely to pick unhealthy snack foods when options are available [10].

Nowadays, food is omnipresent everywhere and there is high consumption of packaged, processed unhealthy foods with increasing snacking frequency. The availability of highly palatable foods enables many opportunities to eat food for the purpose of enjoyment and in the absence of calories, it is crucial to know the role of hedonic hunger on snacking behaviour and food choices that are made. Despite the significance of snacking behaviour and hedonic hunger, there is a lack of comprehensive studies examining their relationships in India. Existing research has focused on defining hedonic hunger, estimating the hedonic hunger rate among young adults, and correlating hedonic hunger with any disease, BMI, obesity or self-control, self-motivation, and emotional eating. No studies so far have examined the role of hedonic hunger on snacking behaviour and food choices. Therefore, conducting a study specifically targeting the Indian population will contribute novel insights to the field and address the existing research gap.

The primary objective of this study was to investigate the relationship between hedonic hunger and snacking behaviour among young adults aged 15-24 years in Vadodara, Gujarat. Secondary objectives include exploring the associations between hedonic hunger and other factors such as emotional eating, body mass index (BMI), and sociodemographic factors. By addressing these objectives, we aim to shed light on the intricate interplay between hedonic hunger, snacking behaviour, and weight-related outcomes in the Indian context.

## Materials and methods

Participants and settings

The aim of the study was to examine the prevalence of hedonic hunger and its interrelationship with snacking behaviour. The study participants consisted of individuals aged 15-24 years residing in urban areas of Vadodara, Gujarat. A simple random sampling technique was used to recruit the participants wherein, participants with any comorbid conditions, chronic illnesses, or eating disorders that could affect appetite were excluded from the study. The sample size of 150 was calculated using Cochran’s formula, considering a prevalence rate of 26% for eating behaviour [11]. A total of 170 participants were initially enrolled in the study. However, 10 participants were excluded due to incomplete information, and an additional 10 participants were excluded because of the presence of comorbidities. The total sample size was 150. 

Design and ethics 

A cross-sectional study was performed on participants who provided informed consent. This study design was utilized to assess the characteristics and relationships within a specific population at a single point in time. Participants were recruited on a voluntary basis, and for individuals below 18 years of age, parental consent was obtained. The study was approved by the Institutional Research Committee (IRC) of Symbiosis School of Health Sciences (SIHS) on September 8, 2020, and the Institutional Ethics Committee (IEC) of Symbiosis International Deemed University, Pune on April 7, 2021. This study was carried out according to the STrengthening the Reporting of OBservational studies in Epidemiology (STROBE) cross-sectional reporting guidelines [12].

Data collection 

A structured questionnaire was administered using online platforms, specifically Google Forms. The data was collected during the second wave of COVID-19 and hence online platforms were chosen for data collection. Study details and procedures were explained to the participants through various means, including Google Meet and WhatsApp video calls. The questionnaire comprised three sections.

Section 1: Anthropometric and Demographic Measures

Since data collection was conducted online, participants self-reported their age, gender, education, occupation, and anthropometric measurements i.e. height (cm) and weight (kg). Body Mass Index (BMI) was calculated for each participant based on height and weight measurements. The BMI was categorized according to the classification provided by the World Health Organization [13]. 

*Section 2: Hedonic Hunger* 

The Power of Food Scale (PFS), a standard questionnaire, was used to assess respondents' hedonic hunger. It evaluates the psychological effects of living in a food-rich environment, presuming that the respondent lives in a setting with enough appealing foods. The PFS provides insight into an individual's appetite-related thoughts and motivations, independent of actual feelings related to the food environment. It was divided into three levels of proximity: food available, food present, and food consumed. Some questions in the questionnaire, such as those involving alcohol or tobacco, were excluded to ensure appropriateness for participants below 18 years of age. The questionnaire included a set of 21 questions presented on a Likert scale ranging from "Don't agree at all" to "Strongly agree"[14]. While specific scoring details were not available, the PFS scores were categorized into tertiles, and a score range was established for analysis purposes.

To assess the factors affecting hedonic hunger, a self-structured questionnaire was prepared using validated questionnaires to study the relation of various factors like meal preparation, physical activity, emotional eating, influence of social media, and appetite levels to hedonic hunger. Considering that hedonic hunger can be influenced by these factors, it was essential to include them in the assessment. The questionnaire consisted of specific subheadings corresponding to each factor, and three questions were included for each factor to maintain uniformity.

*Section 3: Snacking Behaviour* 

Food Frequency Questionnaire (FFQ) is a dietary assessment method used for obtaining dietary data that uses a specific food list to understand the usual diet and relationships between consumption and health outcomes. Participants completed the food frequency questionnaire, which consisted of a specific food list and a frequency response section for reporting their typical dietary intake. The FFQ was adapted from a structured questionnaire, with certain modifications made to align with the study's requirements. Food items and beverages consumed commonly in Gujarat households were added to the checklist. The questionnaire was divided into three parts, namely snacks (43 food items), hot meals (10 food items), and beverages (nine items). The frequency of consumption of commonly consumed snacks like packaged and processed foods, homemade snacks, fruits, soft drinks and energy drinks was recorded along with the portion size. The frequency of consumption varied on a 4-point scale from “once in a day” to never”, and the portion sizes varied from “small to large”. Participants who reported consuming the item “once in a day,” “once in a week,” or “once or twice in a month” were categorized as “Yes,” while those who responded with “never” were categorized as “No”.

Statistical analysis

We analysed the data with Statistical Package for the Social Sciences version 25.0 (IBM Corp., Armonk, NY) with a 95% confidence interval i.e., P <0.05 was considered to be significant for all tests. To avoid any errors, the data was verified and checked. Data is presented as mean ± standard deviation (SD) for continuous variables and frequency and percentage (%) for categorical variables. The Hedonic Hunger scores of the participants were divided into tertiles. A Chi-square test of independence was used to find out if there was a significant association between the three categories of hedonic hunger and various demographic characteristics like gender, occupation, and education of participants. Additionally, this test was used to investigate the relationships between hedonic hunger and other factors, specifically emotional eating, loss of control, lack of willpower, and exercise. The association between hedonic hunger and snacking behaviour, considering a range of healthy and unhealthy food items, was also examined using this test. Pearson correlation test was performed to determine whether there existed a relationship between two continuous variables (hedonic hunger score, weight, BMI) and to identify the strength and direction of that relationship.

## Results

We included data from 150 participants between the ages of 15-24 years in the analysis. Socio-demographic and anthropometric characteristics of young adults have been outlined in Table 1. We observed that the majority of the population belonged to 20-24 years of age (91.3%). A total of 62.7% were females while 37.3% were males. With respect to anthropometric characteristics, 38% of participants were in the normal category for BMI, However, the majority (49.4%) were either in the obese (28.7%) or overweight (20.7%) category. The mean BMI was noted to be 23.52 + 4.92kg/m^2^. The study found no significant associations between socio-demographic variables such as gender and education with hedonic hunger. Similarly, BMI, as an anthropometric measure, did not exhibit a statistically significant association with hedonic hunger. Notably, it's intriguing to observe that the majority of individuals within the normal BMI category (31.6%) fell into the lower levels (0-33) of the hedonic hunger category, contrasting with those who were underweight (26.3%), overweight (22.6%), and obese (18.6%).

**Table 1:** Sociodemographic and anthropometric characteristics of the participants and their association with hedonic hunger (N= 150) *Indicates level of significance at **P < 0.05 , *P < 0.01.

Variable	Total n(%)	Hedonic hunger category n (%)			χ^2 ^value	P-value
		Low (0-33)	Moderate (34-58)	High (>58)		
Gender						
Male	56 (37.3)	14 (25.0)	30 (53.6)	21 (21.4)	0.230	0.891
Female	94 (62.7)	24 (25.5)	47 (50.0)	23 (24.5)		
Occupation						
Student	105 (70)	26 (24.8)	53 (50.5)	26 (24.8)	4.286	0.638
Business	6 (4)	3 (50.0)	3 (50.0)	0 (0.0)		
Service	30 (20)	8 (26.7)	16 (53.3)	6 (20.0)		
Others	9 (6)	1 (11.1)	5 (55.6)	3 (33.3)		
Anthropometry						
BMI categories						
Underweight (<18kg/m^2^)	19 (12.7)	5 (26.3)	7 (36.8)	7 (36.8)	5.761	0.450
Normal (18-22.99 kg/m^2^)	57 (38)	18 (31.6)	30 (52.6)	9 (15.8)		
Overweight (23-24.9 kg/m^2^)	31 (20.7)	7 (22.6)	16 (51.6)	8 (25.8)		
Obese (>25 kg/m^2^)	43 (28.7)	8 (18.6)	24 (55.8)	11 (25.6)		

Table 2 illustrates the association of hedonic hunger with the consumption of specific food items, broadly classified as “Healthy” and “Unhealthy”. The association was found to be significant for healthy food items like rice flakes (χ2 6.629, P 0.036), sprouts (χ2 6.641, P 0.036), salad (χ2 8.812, P 0.017), puffed ragi (χ2 8.824, P 0.012) and musk melon (χ2 6.917, P 0.031). For unhealthy snacks a significant association was only found for roasted salted chana (χ2 6.6004, P 0.049). Based on the findings presented above, a relationship between hedonic hunger and snacking behaviour becomes evident. This observation could potentially be attributed to the prevalence of traditional household snacks in Gujarat, such as poha (rice flakes), chivda (ragi puffed), and fruit or sprout chat, which are widely enjoyed as popular snack items. In all the above cases, it was observed that a greater percentage of participants who had a high level of hedonic hunger did not consume the item, whereas a greater percentage with a low level of hedonic hunger did consume the item. However, consumption of various unhealthy foods was not statistically significant with hedonic hunger; this could be because participants might have diverse preferences for unhealthy foods. This variability can make it challenging to establish a clear and definitive association, especially when contrasted with the availability and popularity of healthy snacks often prepared in households.

**Table 2:** Association of hedonic hunger with snacking behaviour (N=150) *Indicates level of significance at **P < 0.05 , *P < 0.01.

Food Item	Total n(%)	Hedonic hunger category n (%)			χ^2 ^value	P-value
		Low (0-33)	Moderate (34-58)	High (>58)		
Healthy						
Rice flakes						
Yes	124 (82.7)	32 (25.8)	68 (54.8)	24 (19.4)	6.629	0.036*
No	26 (17.3)	6 (23.1)	9 (34.6)	11 (42.3)		
Sprouts						
Yes	121 (80.7)	33 (27.3)	65 (53.7)	23 (19.0)	6.641	0.036*
No	29 (19.3)	5 (17.2)	12 (41.4)	12 (41.4)		
Salad						
Yes	129 (86.0)	35 (27.1)	69 (53.5)	25 (19.4)	8.182	0.017*
No	21 (14.0)	3 (14.3)	8 (38.1)	10 (47.6)		
Muskmelon						
Yes	92 (61.3)	24 (26.1)	53 (57.6)	15 (16.3)	6.917	0.031*
No	58 (38.7)	14 (24.1)	24 (41.4)	20 (34.5)		
Ragi Puff						
Yes	58 (38.7)	17 (29.3)	35 (60.3)	6 (10.3)	8.924	0.012*
No	92 (61.3)	21 (22.8)	42 (45.7)	29 (31.5)		
Unhealthy						
Potato Chips						
Yes	136 (90.7)	33 (24.3)	71 (52.2)	32 (23.5)	0.897	0.639
No	14 (9.3)	5 (35.7)	6 (42.9)	3 (21.4)		
Ready to eat sticks						
Yes	111 (74.0)	26 (23.4)	59 (53.2)	26 (23.4)	0.892	0.640
No	39 (26.0)	12 (30.8)	18 (46.2)	9 (23.1)		
Vegetable Puff						
Yes	122 (81.3)	29 (23.8)	61 (50.0)	32 (26.2)	3.206	0.201
No	28 (18.7)	9 (32.1)	6 (57.1)	3 (10.7)		
Instant Noodles						
Yes	142 (94.7)	37 (26.1)	74 (52.1)	31 (21.8)	3.440	0.179
No	8 (5.3)	1 (12.5)	3 (37.5)	4 (50.0)		
Pasta						
Yes	138 (92.0)	34 (24.6)	73 (52.9)	31 (22.5)	1.712	0.425
No	12 (8.0)	4 (33.3)	4 (33.3)	4 (33.3)		
Bread Butter Jam						
Yes	139 (92.7)	36 (25.9)	69 (49.6)	34 (24.5)	2.330	0.312
No	11 (7.3)	2 (18.2)	8 (72.7)	1 (9.1)		
Roasted salted Chana						
Yes	120 (80.0)	33 (27.5)	64 (53.3)	23 (19.2)	6.044	0.049*
No	30 (20.0)	5 (16.7)	13 (43.3)	12 (40.0)		

The association between hedonic hunger and emotional factors and exercise that can influence hedonic hunger score has been enumerated in Table 3. Hedonic hunger was significantly associated with all four factors (p<0.05). A notable percentage of participants, specifically those who reported engaging in emotional eating (31.3%), feeling a loss of control when satisfying cravings (32.7%), lacking the willpower to control cravings (32.7%), and interestingly, those with infrequent exercise habits (26.3%), also indicated a high level of hedonic hunger. These findings suggest that a significant portion of individuals tend to turn to food when experiencing emotional states such as stress, anxiety, depression, or sadness, and they are more inclined to select palatable foods.

**Table 3:** Association between hedonic hunger and various other factors (N=150) *Indicates level of significance at **P < 0.05 , *P < 0.01.

Variable	Hedonic hunger category n (%)			χ^2 ^value	P-value
	Low (0-33)	Moderate (34-58)	High (>58)		
Emotional Eating					
Yes	19 (22.9%)	38 (45.8%)	26 (31.3%)	6.639	0.036*
No	19 (28.4%)	39 (58.2%)	9 (1.4%)		
Losing control to satisfy craving					
Yes	7 (13.5%)	28 (53.3%)	17 (32.7%)	7.514	0.023*
No	31 (25.3%)	49 (50%)	18 (18.4%)		
No willpower to control cravings					
Yes	7 (13.5%)	28 (53.8%)	17 (32.7%)	7.514	0.023*
No	31 (31.6%)	49 (50%)	18 (18.4%)		
Exercise					
Yes	20 (19.4%)	56 (54.4%)	27 (26.3%)	6.600	0.043*
No	18 (38.3%)	21 (41.7%)	8 (17%)		

Table 4 illustrates the correlation between hedonic hunger score, BMI, and weight. No correlation existed between BMI and hedonic hunger score. However, a positive correlation between the weight of the participants and their hedonic hunger score was observed (r-value= 0.170, P 0.038). The absence of a correlation between BMI and hedonic hunger, alongside the presence of a correlation between weight and hedonic hunger, can be attributed to BMI's inherent limitations and the dynamic nature of weight as an independent variable.

**Table 4:** Pearson’s correlation between hedonic hunger, BMI, and weight of the participants (N=150) *Indicates level of significance at **P < 0.05 , *P < 0.01.

Variable	r-value	P-value
BMI(Kg/m2)	0.056	0.495
Weight (Kg)	0.170	0.038*

## Discussion

The above study aimed to assess the impact of hedonic hunger on snacking behaviour in adolescents between the ages of 15 and 24 years residing in Vadodara, Gujarat. The majority (91.3%) of the study participants belonged to the age category of 20-24 years, with a recorded mean age of 22.05 + 1.69 years. The nutrition assessment data briefly presented that the majority of the individuals were in the normal category of BMI (38%), followed by the obese category (28.7%). The mean BMI and mean weight were also noted to be on the higher side (23.52kg/m2, 64kg). Regarding other factors such as occupation and education, most of the participants were students (70%) pursuing undergraduate degrees (58%). The objectives of the study have been fulfilled and are complemented by an analysis of the association between various variables.

Hedonic Hunger is now considered the new measure of appetite that can be defined as the desire to consume more appetising food items in the absence of physical hunger [14,15]. The power of food scale assessment showed that hedonic hunger was moderately present in 51.3% of the individuals along with 23.3% of individuals who showed severe signs of hedonic hunger and were thought to have stronger stimulus towards consumption of delicious foods rich in salt, sugar, and fat. The present study also analysed the relationship of hedonic hunger with gender and it was seen that hedonic hunger is more prevalent in females (24.5%) compared to males (21.4%) although the association was not significant (P 0.891). A similar observation was made by Ewoldt J, 2012, which showed that the presence of hedonic hunger is associated with gender and is more prevalent in females compared to males (mean score=2.62 vs 2.48) [16]. Ewoldt J also briefly associated hedonic hunger with age and concluded that hedonic hunger was more prevalent in young adults (18-29 years) compared to older age groups (+62 years) [16]. 

Snacking can be defined as the consumption of food and drinks including items like chips, chocolates, soft drinks, etc. Studies worldwide have shown high rates of snacking behaviour among young people, especially college-going students [17]. With respect to snacking behaviour, the majority of the participants indulged in snacking on a frequent basis and consumed various food items. Previous studies have suggested a potential relationship between hedonic hunger and snacking behaviour. To determine the role of hedonic hunger on snacking behaviour, a list of food items including refined, processed, and nutritious snacks was compared against hedonic hunger categories. The association was found to be significant for food items like rice flakes (P 0.036), sprouts (P 0.036), salad (P 0.017), ragi puff (P 0.012), roasted salted chana (P 0.049) and muskmelon (P 0.031). In a study conducted by Ello-Martin et al., 2007, the recommendation to include water-rich foods such as fruits and vegetables in a reduced-fat diet proved to be more effective in managing feelings of hunger, achieving weight loss, and improving certain physiological markers compared to solely following a reduced-fat diet [18]. While reducing fat intake is commonly recommended to decrease the energy density of the diet, incorporating foods with high water content can provide additional benefits in terms of reducing energy density. Similarly, a Danish study found that irregular breakfast, lunch, and evening meal consumption among adolescents was associated with a low frequency of fruit and vegetable intake among adolescents aged 11-15 years [19]. The consumption of other palatable foods like instant noodles, pasta, and vegetable puffs was also reported on the higher side (21.8%, 22.5%, and 26.2%) for those who reported high hedonic hunger scores respectively.

Living in an obesogenic environment is one of the strongest factors that can induce hedonic hunger, but there are many environmental and external factors that can induce hedonic hunger and increase food consumption which mainly include portion size, salience of the food, social aspects of food, lifestyle, peer influence, number of meals consumed, and duration of the meal, physical activity, sleep and food cravings. Various eating atmospherics like lighting, odour, ambience, noise, and music are also likely to influence immediate food consumption. A self-administered questionnaire on factors of hedonic hunger was administered to all the participants. A significant association was observed between hedonic hunger and emotional eating (P 0.036), losing control to satisfy craving (P 0.023), no willpower to control craving (P 0.023), and exercise (P 0.043). Consistent with this assertion, a study conducted by Mason et al., 2020, briefly explained the relationship between hedonic hunger and emotional eating [20]. Changes in negative urgency which is the tendency to act impulsively when experiencing negative emotions predicted increased hedonic hunger. Horwath et al., 2020, found that self-control significantly weakened the positive relationships between hedonic hunger and factors such as overeating frequency, snacking frequency, and consumption of palatable foods [21]. Individuals who possess both a heightened sensitivity to the easy accessibility of appetizing foods and high levels of self-control demonstrate lower frequencies of overeating and snacking, including reduced consumption of appetising or palatable foods, compared to individuals with lower levels of self-control. According to a review by Jarry LC, 2016, engaging in acute exercise at a specific intensity threshold induces consistent perceptual and hedonic alterations in the general population including immediate suppression of hunger, regulation of thirst related to maintaining proper hydration, heightened palatability of salt and sugar-rich foods, and reduced perception and appeal of sourness [22]. In our study, individuals involved in a certain type of physical activity experienced more hedonic hunger compared to those who were not involved in any kind of physical activity (26.3% vs. 17%). However, more research is needed is needed to determine the exact role and mechanism of exercise on hedonic hunger.

Hedonic hunger can be a significant moderating factor in the presence of obesity. A positive correlation was found between hedonic hunger scores and the weight of the participants (P 0.039). However, no significant association was observed between BMI and the hedonic hunger scores of the participants. In contrast to these findings, a study conducted by Ribeiro et al., 2018 reported a weak association between hedonic hunger and BMI [23]. 

The strength of this study is that it provides a unique contribution by examining the relationship between hedonic hunger and snacking behaviour in adolescents from Vadodara, Gujarat, which has not been explored before. By focusing on this specific population and location, the study offers valuable insights into the factors that are associated with hedonic hunger. However, the study does have limitations. One limitation is that the study relies on self-reported data, which introduces the possibility of response bias. Participants' self-reporting of snacking behaviour, anthropometric measures, hedonic hunger, and other factors may be subject to memory errors, social desirability bias, or inaccurate recall, potentially affecting the reliability of the results. Additionally, the cross-sectional design of the study limits the ability to establish causality. Lastly, the study was conducted in a specific geographic location, restricting the generalizability of the findings to other regions or cultural contexts. Cultural and environmental factors could influence hedonic hunger and snacking behaviour differently in other populations, necessitating caution when applying the study's results to diverse settings. These limitations highlight the need for further research. Future studies employing diverse samples, longitudinal designs, and more robust methodologies can enhance our understanding of the complex relationship between hedonic hunger and snacking behaviour, enabling more targeted interventions and recommendations, especially for young adults. 

## Conclusions

The concurrent rise in the abundance of palatable snack foods and the rate of increasing cases of obesity necessitates a deeper understanding of the pathways that interlink them. This study investigated the impact of hedonic hunger on snacking behaviour in young adults aged 15-24 years in Vadodara, Gujarat. Predominantly, the participants fell within the 20-24 age group, with a majority being females. Among the participants, most displayed a normal BMI, followed by those categorized as obese. Furthermore, the study reported the presence of moderate hedonic hunger among the participants revealing a significant association between hedonic hunger and specific food items. Notably, emotional eating, losing control, no willpower to control cravings, and exercise showed significant associations with hedonic hunger. A positive correlation between hedonic hunger scores and weight implies the relevance of hedonic hunger in snacking behaviour and its potential influence on weight-related factors among young adults. Further research is warranted to explore the mechanisms underlying the relationship between hedonic hunger and obesity.
